# Supplementary material for: Cancer Associated Macrophage-like Cells Are Prognostic for Highly Aggressive Prostate Cancer in Both the Non-Metastatic and Metastatic Settings
Source: Cancers (Basel). 2023 Jul 22;15(14):3725. doi: 10.3390/cancers15143725 (PMC10378487; doi:10.3390/cancers15143725)
Supplement: Supplementary file 1 [file cancers-15-03725-s001.zip › cancers-2454659-supplementary.pdf]

Institution	No. of Patients (% Total)	No. Non-Metastatic (% Total | % Non-Mets)	No. Metastatic (% Total | % Mets)
Fox Chase Cancer Center	10 (11%)	6 (7% | 12%)	4 (4% | 10%)
Mayo Clinic Cancer Center	3 (3%)	2 (2% | 4%)	1 (1% | 2%)
Memorial Sloan Kettering Cancer Center	17 (18%)	1 (1% | 2%)	16 (17% | 38%)
Northwestern University	25 (28%)	24 (27% | 48%)	1 (1% | 2%)
Oregon Health and Science University	37 (40%)	17 (19% | 34%)	20 (21% | 48%)

Supplementary Table S1: Distribution of patient recruitment by institution.

Multivariate Variable	PFS p value	OS p value
Age ≥70	>0.500	**0.041**
pT ≥2	0.486	0.100
pN ≥1	**0.011**	**0.028**
pM ≥1	**0.015**	0.254
Gleason ≥8	**0.048**	0.066
BL PSA ≥50ng/mL	**0.007**	0.060
CTCs ≥1 cells	>0.500	0.169
BL CAMLs ≥3 cells	0.167	>0.500
BL CAML ≥50µm	**0.002**	**0.006**

Supplementary Table S2: Multivariate analysis comparing all known significant clinical variables. Wilcoxon t-test was performed for all known clinical variables (ie. Age, race, treatment type) as predictors of survival prior to multivariate analysis; statistically significant predictors of worse outcome were selected for multivariate analysis.

Pathological Stage (n)	CTC (%)	EMT (%)	CAML (%)
Stage I (n=14)	1 (7%)	8 (57%)	8 (57%)
Stage II (n=28)	4 (14%)	13 (46%)	23 (82%)
Stage III (n=8)	3 (38%)	3 (38%)	8 (100%)
Stage IV (n=40)	11 (28%)	10 (25%)	32 (80%)

Supplementary Table S3: Frequency of cancer-associated circulating cells/ 7.5mL blood in each patient by prostate cancer pathological stage

a. ≥50μm CAML Frequency in Patients Based on CTC Presence

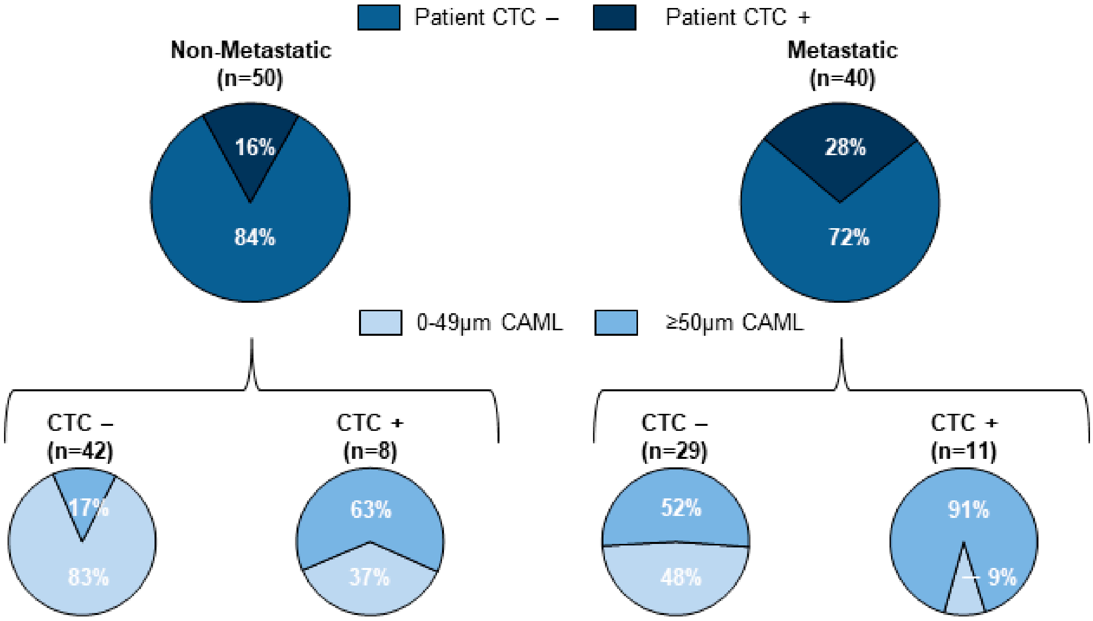

b. Comparing CTC Positivity and CAML ≥50μm Presence to No CTC and CAML ≥50μm

Comparison	ANOVA p-value
Non. vs. Mets CTC + (8 vs. 11)	p=0.188
Non. CTC+ and CAML ≥50μm+ (8 vs. 42)	**p=0.005**
Mets CTC+ and CAML ≥50μm+ (11 vs. 29)	**p=0.022**

c. Fisher's Exact Test Correlating CTC Positivity to CAML ≥50μm Presence

Patient Population	Fisher's p-value
All patients	**p<0.001**
Non-Metastatic	**p=0.014**
Metastatic	**p=0.030**

Supplementary Figure S1: a. Comparison of CTC presence and ≥50μm CAML frequency. Frequency of CTC presence and engorged CAMLs in circulation was examined. **b. ANOVA to Determine Statistical Differences in CTC Presence and ≥50μm CAML.** Single factor ANOVA was used to determine if CTC and engorged CAML presence is statistically different compared to patients who are CTC negative with engorged CAMLs. **c. Contingency Testing.** Fisher's exact test was performed to determine if there is a statistical relationship between CTC positivity and engorged CAML presence.

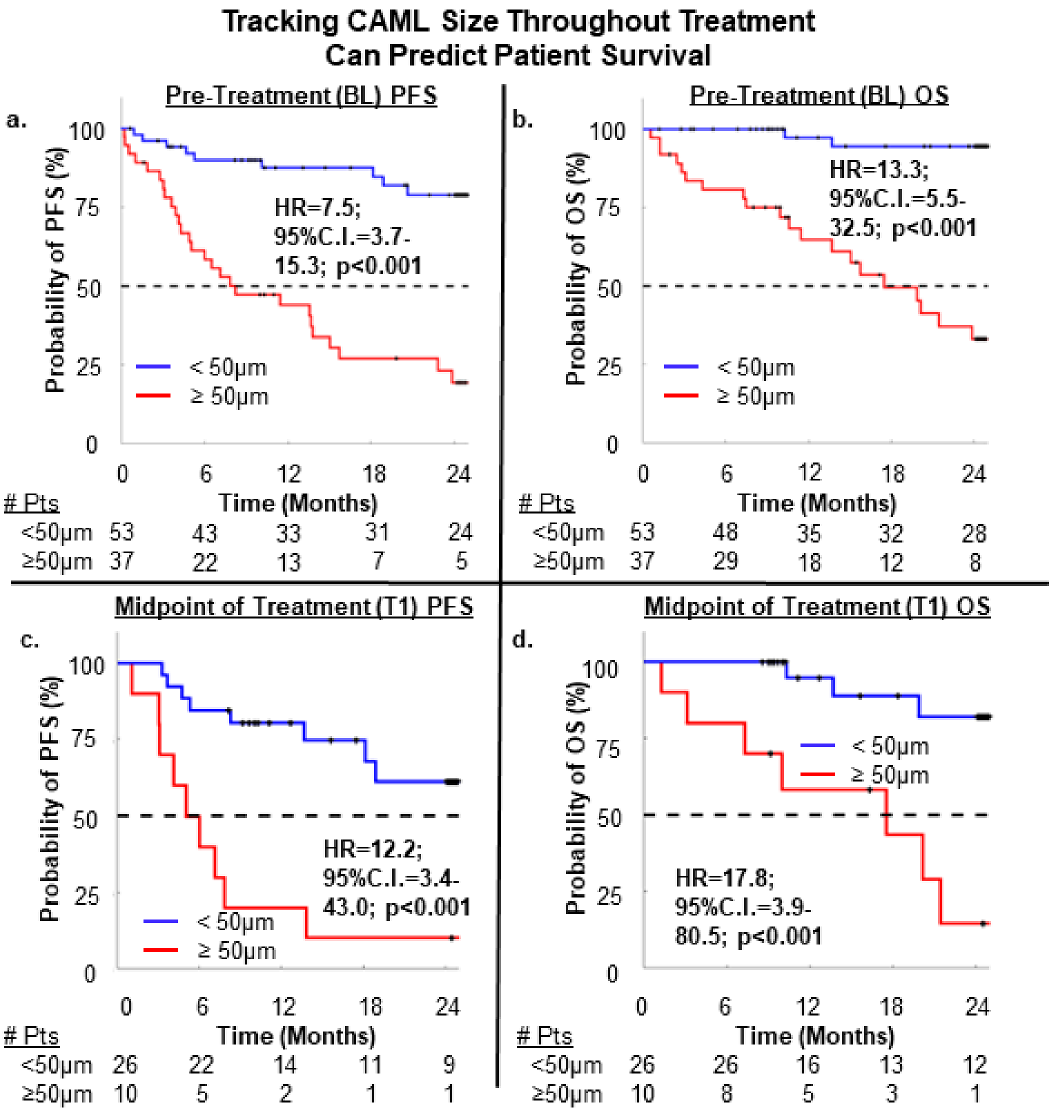

Supplementary Figure S2: Kaplan-Meier graphs of PFS and OS Based on Pre-induction of therapy or Post Induction of therapy (T1) based on CAML Size. a. PFS BL all samples. **b.** OS BL all samples. **c.** PFS T1 samples. **d.** OS T1 samples.

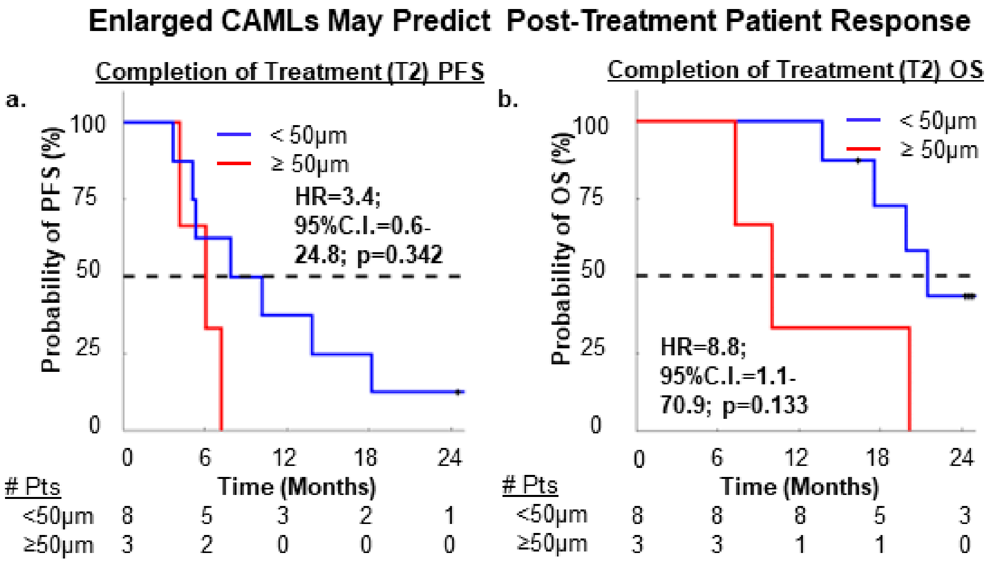

Supplementary Figure S3: Kaplan-Meier graphs of PFS and OS Based on CAML Size at the first blood draw post-completion of treatment (T2). a. PFS T2 samples. **b.** OS T2 samples. All T2 data for analysis was performed solely on metastatic PCa patients due to lack the of patient samples in the non-metastatic setting.

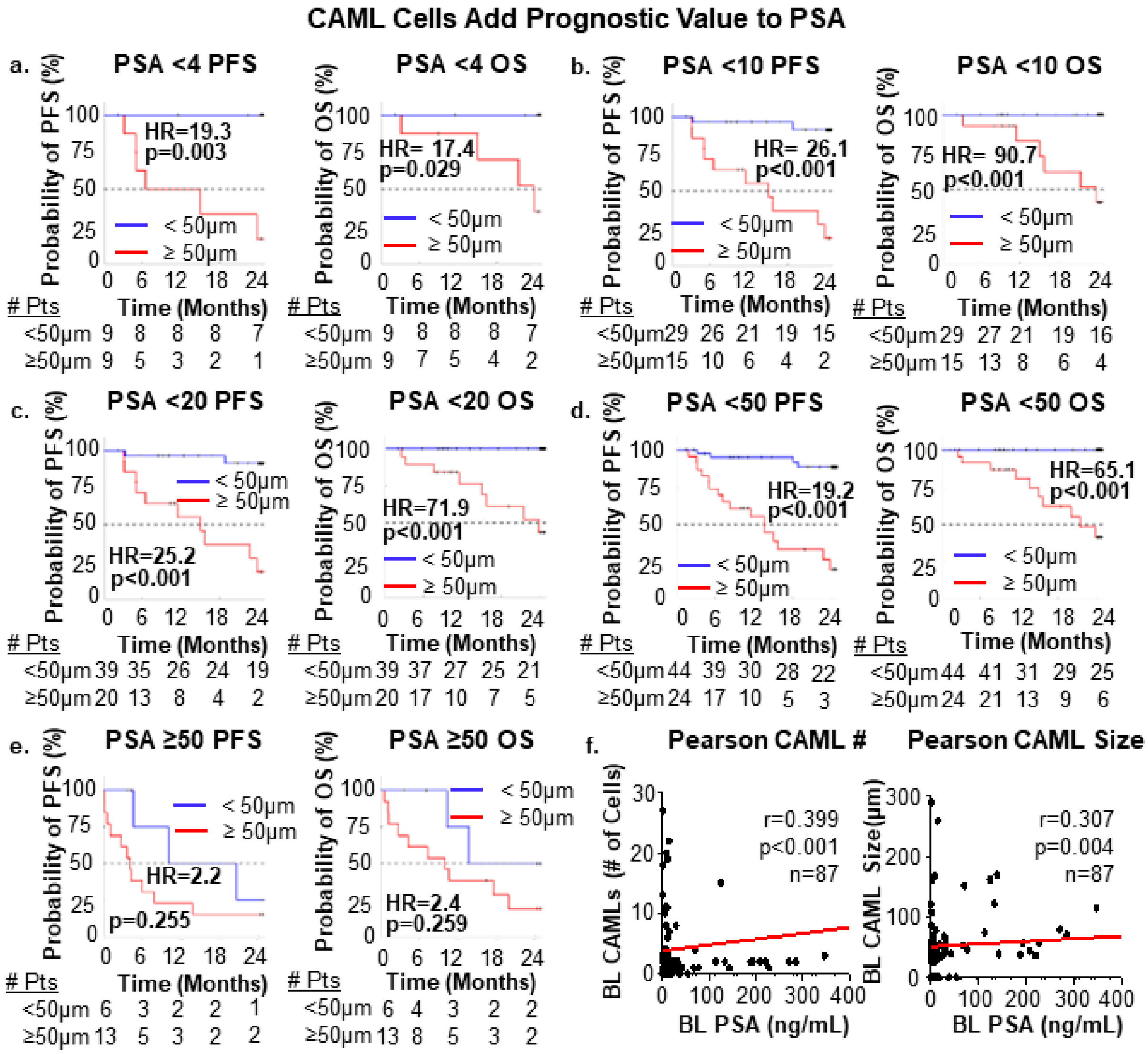

Supplementary Figure S4: Kaplan-Meier graphs portraying the additive prognostic value of BL CAML size to BL PSA for stratifying patient PFS and OS. To determine the additive prognostic value of CAML screening in combination with PSA before a patient start treatment, we selected patients with low levels of PSA (<4ng/mL), within the "gray zone" for active surveillance (<10ng/mL), as well as medium-high (<20ng/mL, <50ng/mL), and very high (≥50ng/mL) PSA at diagnosis. We found that engorged CAMLs, in combination with low to medium-high PSA can better stratify patient survival than PSA alone. **a.** All patients with PSA <4ng/mL at BL. **b.** All patients with PSA <10ng/mL at BL. **c.** All patients with PSA <20ng/mL at BL. **d.** All patients with PSA <50ng/mL at BL. **e.** All patients with PSA ≥50ng/mL at BL. We then sought to determine if there is any correlation between rising PSA and increasing CAML cells in circulation as well as rising PSA to increasing CAML cell size. We found that **f.** there is a statically significant, weak positive correlation between the number of CAMLs in circulation and rising PSA, as well as statically significant, weak positive correlation of increasing CAML size with rising PSA. Two patients were not graphed in the # of CAMLs correlation to better visualize the regression curve. Three patients were not graphed in the CAML size correlation to better visualize the regression curve.

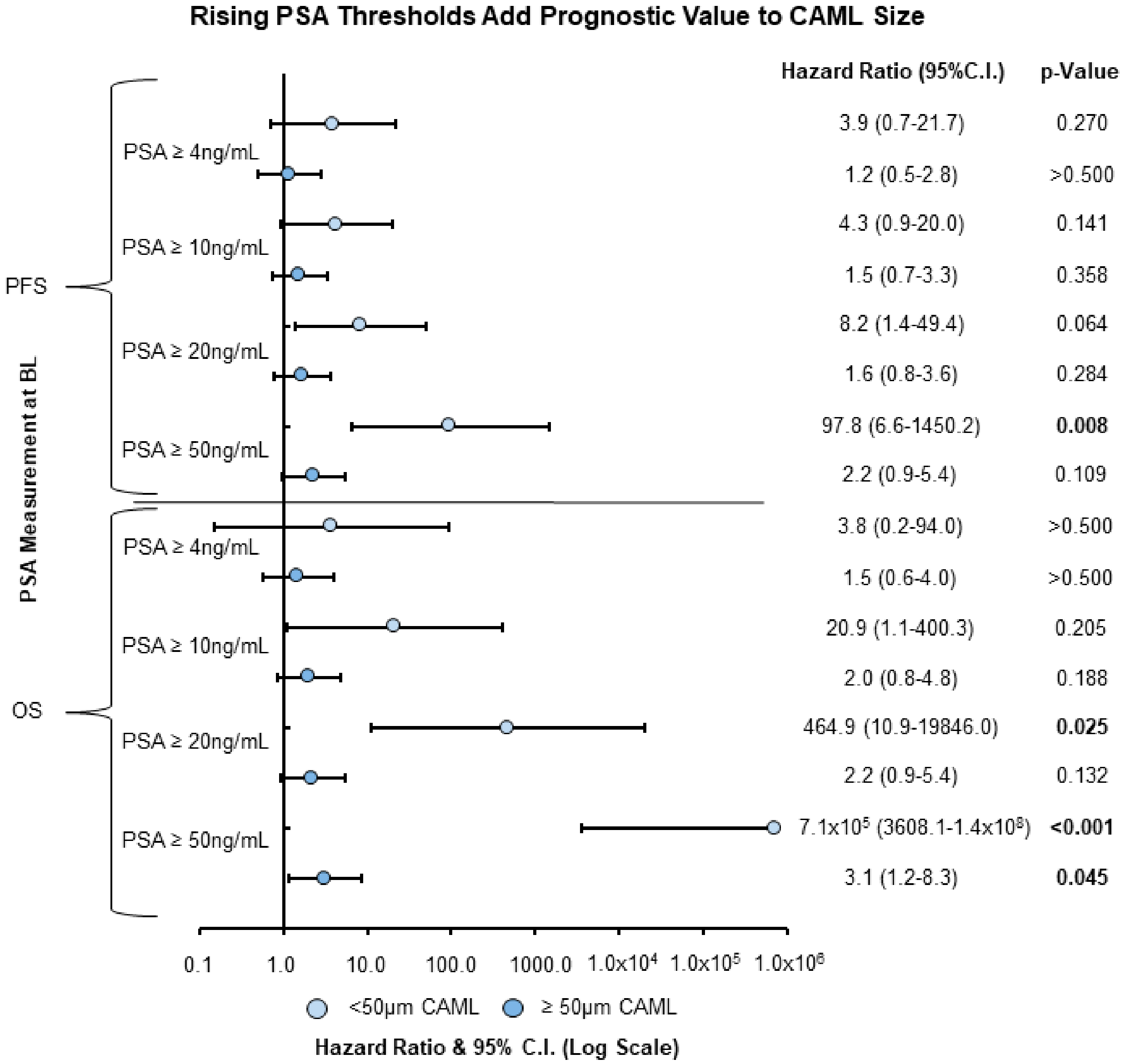

Supplementary Figure S5: Forest plot combining PSA with CAML size to prognosticate patient survival. To determine if PSA can complement CAML size in prognosticating PFS and OS, we looked at different thresholds of PSA in patients with either <50µm CAMLs or ≥50µm CAMLs at BL. Kaplan-Meier analyses compared PSA thresholds greater than or equal to designated cut-offs versus lower PSA levels. Patients with CAMLs <50µm had improved survival for PSA thresholds ≥ 50ng/mL for PFS and PSA ≥ 20ng/mL and ≥ 50ng/mL for OS. Patients with CAMLs ≥ 50µm only showed worse OS when PSA ≥ 50ng/mL.
